# Rifampicin resistance mutations in the 81 bp RRDR of *rpo*B gene in *Mycobacterium tuberculosis* clinical isolates using Xpert MTB/RIF in Khyber Pakhtunkhwa, Pakistan: a retrospective study

**DOI:** 10.1186/s12879-016-1745-2

**Published:** 2016-08-12

**Authors:** Irfan Ullah, Aamer Ali Shah, Anila Basit, Mazhar Ali, Afsar khan, Ubaid Ullah, Muhammad Ihtesham, Sumaira Mehreen, Anita Mughal, Arshad Javaid

**Affiliations:** 1 Department of Microbiology, Faculty of Biological Sciences, Quaid-i-Azam University, Islamabad, Pakistan; 2 Programmatic Management of Drug resistant TB Pulmonology, Lady Reading Hospital Peshawar, Peshawar, Pakistan; 3 Department of Biotechnology and Microbiology, Sarhad University of Science and Information technology Peshawar, Peshawar, Pakistan

**Keywords:** RRDR, RNA polymerase B gene, MDR-TB, Xpert MTB/RIF

## Abstract

**Background:**

Multi-drug resistant tuberculosis (MDR-TB) is a major public health problem especially in developing countries. World Health Organization (WHO) recommends use of Xpert MTB/RIF assay to simultaneously detecting *Mycobacterium tuberculosis* (MTB) and rifampicin (RIF) resistance. The primary objective of this study was to determine the frequency of MDR-TB in patients suspected to have drug resistance in Khyber Pakhtunkhwa. The frequency of probes for various *rpo*B gene mutations using Xpert MTB/RIF assay within 81 bp RRDR (Rifampicin Resistance Determining Region) was the secondary objective.

**Methods:**

A total of 2391 specimens, received at Programmatic Management of Drug Resistant TB (PMDT) Unit, Lady Reading Hospital (LRH) Peshawar, Pakistan, between October 2011 and December 2014, were analyzed by Xpert MTB/RIF test. MTB positive with rifampicin resistance were further analyzed to first line anti-mycobacterial drug susceptibility testing (DST) using middle brook 7H10 medium. The data was analyzed using statistical software; SPSS version 18.

**Results:**

Out of 2391 specimens, 1408 (59 %) were found positive for MTB and among them, 408 (29 %) showed rifampicin-resistance with four different *rpo*B gene mutations within 81 bp RRDR. The frequency of various probes among RIF-resistant isolates was observed as: probe E, in 314 out of 408 isolates; B, 44 out of 408; A, 5 out of 408; D, 34 out of 408; and probe C was observed among 6 out of 408 RIF-resistant isolates. The probe A&B and E&D mutation combination was found in only 1 isolate in each case, while B&D mutation combination was detected among 3 out of 408 RIF-resistant isolates.

**Conclusions:**

Hence, it is concluded from our study on a selected population, 29 % of patients had MDR-TB. Probe E related mutations (also known as codon 531and 533) were the most common *rpo*B genetic mutation [314 (77 %)], acknowledged by Xpert MTB/RIF assay. Least mutation was detected within the sequence 511 (1.2 %).

## Background

Multi-drug resistant tuberculosis (MDR-TB), is defined as a form of TB infection caused by *Mycobaterium tuberculosis* that is resistant to treatment with at least two of the most powerful first-line anti-TB drugs, isoniazid and rifampicin [1]. Rifampicin (RIF) resistance is of specific epidemiological importance and a valuable surrogate marker for MDR-TB strains as more than 90 % RIF resistant strains are also resistant to isoniazid [2, 3]. Introduction of Xpert MTB/RIF assay (Cepheid, USA) has revolutionized the diagnosis of TB by simultaneously detecting MTB and rifampicin resistance [4, 5]. Rifampicin is one of the key first-line anti-tuberculosis drug, which inhibits DNA-directed ribonucleic acid synthesis of MTB proteins by binding to the β-subunit of bacterial DNA dependent RNA polymerase enzyme (*rpo*B) protein. In general, *rpo*B mutations is found in 95–97 % of RIF-resistant MTB strains worldwide and these mutations are typically located in a region at the 507–533 amino acid residuals (81 bp) within the *rpo*B genetic factor, that is usually referred to as Rifampicin Resistant Determinant Region (RRDR) [6].

Pakistan ranks 5th among 22 high TB burden countries and 4th among MDR-TB countries. WHO report showed that the prevalence of MDR-TB was 4 % and 35 % among all new and previously treated TB cases, respectively [1]. The biological features of bacteria as well as drug resistance typically vary in different geographical areas [7–9]. Data on prevalence of *rpo*B gene mutations in Pakistan is limited, so this study is aimed to provide reference line data on these mutations using Xpert MTB/RIF assay. The detection of *rpo*B gene mutations is most important for accurate diagnosis of RIF resistance in MTB strains [10]. The current study was aimed to investigate the frequency of RIF-resistant MTB strains in specimens collected from TB presumptive patients in Khyber Pakhtoonkhwa, Pakistan and also mutations at RRDR within the *rpo*B gene of RIF-resistant MTB using Xpert MTB/RIF assay.

## Methods

We studied 2391 patients in this study. Each patient was required to provide one sputum specimen.

### Clinical specimens

Total numbers of 2391 specimens from any drug resistant TB presumptive patients were received at PMDT, LRH between October 2011 and December 2014. All these specimens were investigated for detection of MTB as well as resistance to the first-line anti-TB drug, Rifampicin using Xpert MTB/RIF. The referrals of these specimens were according to the guidelines of NTP for Xpert MTB/RIF.

### Criteria for testing on Xpert MTB/RIF

The following criteria was considered for testing specimens on Xpert MTB/RIF: 1. All smear positive new cases that remain positive by the end of 2nd month of treatment; 2. Failure of Cat-I and Cat-II; 3. Re-treated pulmonary cases both smear positive and smear negative;4. Contact with MDR-TB patients; and 5. TB/HIV co-infection cases were considered for testing through Xpert MTB/RIF.

**Fig. 1:**
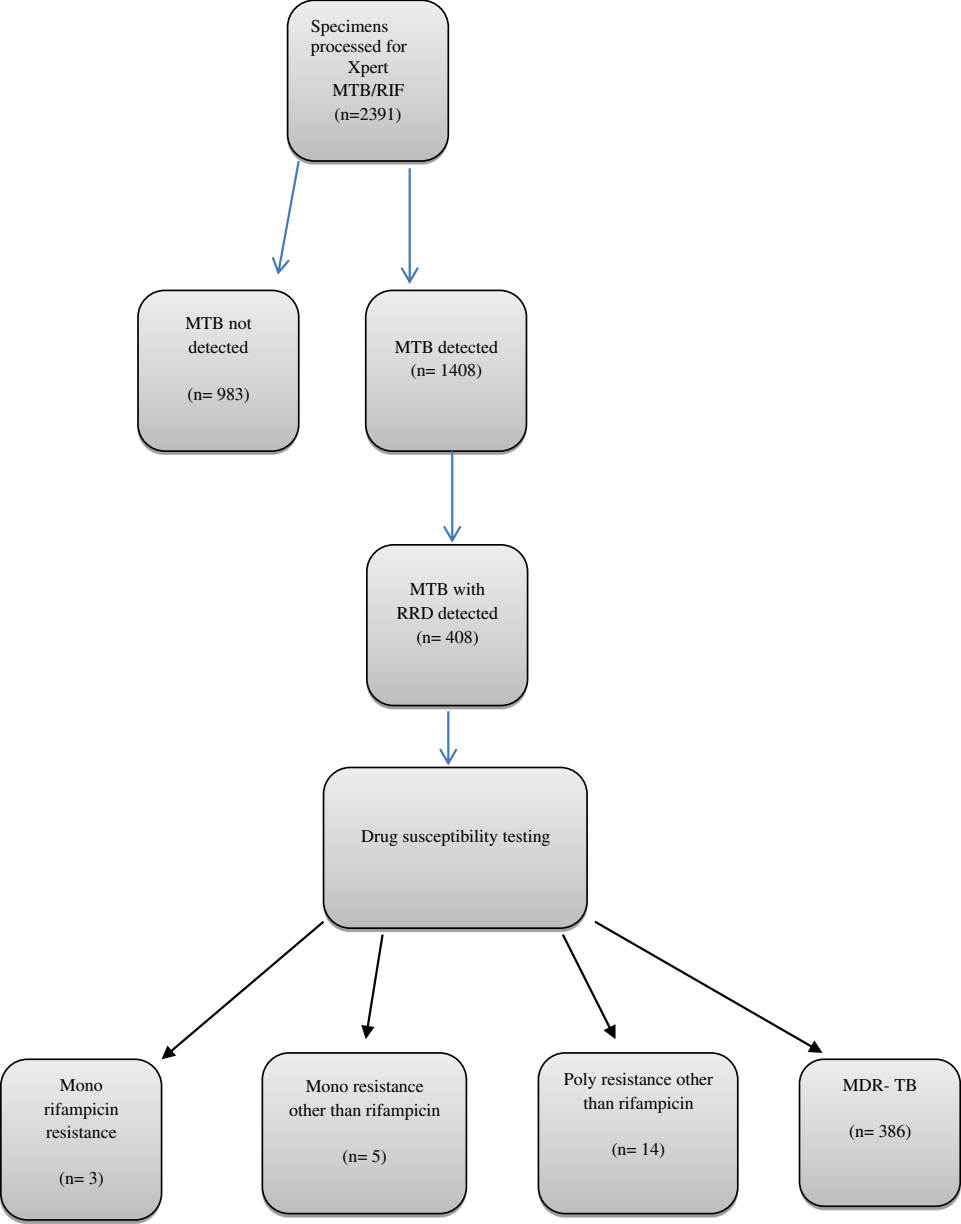
Flow chart of specimens’ analysis (submitted separately via online)

Important details and specimen characteristics (volume and consistence) were recorded in laboratory data assortment forms and later on transferred to access restricted computer-based database. These specimens included sputa, cerebral spinal fluid (CSF), pleural fluid, pus, ascetic fluid, pericardial fluid and bronchial wash (Table 1). TB diagnostics requests ranged from microscopy, culture and Xpert test. In addition to routine analysis, we also performed DST on all MTB positive and rifampicin resistant detected (RRD) cases that were identified by Xpert MTB/RIF test as shown in Fig 1.

**Table 1:** Breakdown of samples in extra pulmonary Tuberculosis

Sample name	Frequency (%)
Sputum	2272 (95)
Bronchial Wash	54 (2.2)
Ascetic fluid	23 (1)
CSF	8 (0.3)
PUS	23 (1)
Pericardial fluid	11 (0.5)
Total	2391

### Drug susceptibility testing

Drug susceptibility testing was performed using the standard agar proportion method on enriched Middle brook 7H10 medium (BBL, Bechton Dickinson and Company, New Jersey) at the following drug concentration: Isoniazid 0.2 and 1 μg/ml, rifampicin 1 and 5 μg/ml, ethambutol 5 and 10 μg/ml and streptomycin 2 and 10 μg/ml [11, 12]. *Mycobacterium tuberculosis* H37Rv (sensitive to all anti-tuberculosis drugs) was used as reference control strain. The drugs were used at critical concentrations.

### MTB/RIF assay

Non-sterile clinical specimens were processed by the standard N-acetyl-L-cysteine–NaOH technique. [13] Smears were prepared by the auramine-rhodamine acid-fast staining technique. The MTB/RIF assay was done as described previously [4, 14]. N-acetyl-L-cysteine–NaOH was added to the clinical specimens at a 3:1 ratio, for decontamination in a closed specimen. The container was manually agitated 15-min period at room temperature. 2 ml of inactivated specimen was transferred to the Xpert test cartridge and tested according to manufacturer's guide [4, 14].

### Statistical analysis

The data was analyzed by using Statistical Package for Social Sciences (SPSS 18).

## Results

### Xpert® MTB/RIF assay

Out of RRD cases, 42 (3 %) were new and 366 (26 %) previously treated cases. Females were 243 (59.6 %) and males 165 (40.4 %). Maximum number of RRD patients was found in the age group 15–24 years (38.5 %) as shown in Table 2.

**Table 2:** Detection of mutation in *rpo*B gene of rifampicin in rifampicin resistance detected (RRD) cases with different demographic representation

Demography	Probe types								Total (%)
	A	B	C	D	E	A&B	B&D	E&D	
Gender									
Female	3	25	4	22	187	1	0	1	243 (59.6)
Male	2	19	2	12	127	0	3	0	165 (40.4)
History									
New patients	1	1	1	4	35	0	0	0	42 (10.3)
Previously treated patients	4	43	5	30	279	1	3	1	366 (89.7)
Age categories (years)									
<14	0	2	0	3	24	0	0	0	29 (7.1)
15- 24	0	14	3	12	125	1	2	0	157 (38.5)
25- 34	3	11	1	7	82	0	0	0	104 (25.5)
35- 44	2	9	0	2	36	0	0	0	49 (12)
45- 54	0	3	1	1	25	0	0	1	31 (7.6)
55- 64	0	3	1	7	19	0	0	0	30 (7.3)
64<	0	2	0	2	3	0	1	0	8 (2)

Among 2391 specimens tested by Xpert MTB/RIF, MTB was detected in 1408 (58.9 %) specimens. Out of total MTB cases, 408 (29 %) were detected to have RIF-resistance. The resistance was given by four totally different *rpo*B gene mutations within the 81 bp-RRDR of MTB. These were detected by A, B, C D, E, A&B, B&D and E&D probes. The probe frequency that reflects RIF-resistance was observed as follows: probe A frequency was 1.2 %; B, 10.8 %; C, 1.5 %; D, 8.3 %; and probe E frequency was highest (77 %) among all. Mutation combination of probe A&B and E&D was 0.25 %, while a B&D mutation combination was 0.7 % (Table 2).

408 RRD cases, checked by Xpert MTB/RIF were further subjected to DST. Out of 408 cases, 19 (4.6 %) were failed to show RIF-resistance, 5 (1.2 %) mono resistant and 14 (3.4 %) showed poly resistance to first-line anti-TB drugs other than rifampicin, while 386 (94.6 %) cases were found to be MDR on DST (Table 3). 252 (61.6 %) cases were resistant to all first-line anti-TB drugs while 386 (94.6 %) cases of rifampicin resistant were also found resistant to isoniazid.

**Table 3:** Pattern of anti-TB drugs susceptibility testing against RRD cases (n = 408) by Xpert MTB/RIF

Anti-TB Drugs	Resistance (n)	Resistance (%)
H	3	(0.73)
R	3	(0.73)
S	2	(0.5)
ES	2	(0.5)
HE	3	(0.73)
HS	2	(0.5)
HZ	2	(0.5)
HR	3	(0.73)
HEZ	2	(0.5)
HRE	2	(0.5)
HRZ	34	(8.3)
HEZS	3	(0.73)
HREZ	72	(17.6)
HRZS	23	(5.6)
HREZS	252	(61.6)

*H* isoniazid, *R* rifampicin, *S* streptomycin, *E* ethambutol, *Z* pyrazinamide

## Discussion

The genetic basis of anti-tuberculosis drug resistant MTB isolates has been widely studied worldwide and commonly believed to be caused by point mutations in some important genes like *rpo*B, *kat*G, *rps*L, *emb*B etc. [6]. Meanwhile globally MDR-TB is big threat to human especially in developing countries, therefore in the present study we use Xpert MTB/RIF to check drug resistant isolates of MTB for mutation in the *rpo*B gene of rifampicin as more than 90 % strains are also resistant to isoniazid [2, 3]. Khyber Pakhtoonkhwa is one of the four provinces of Pakistan, that share a common border with Afghanistan, and over 30 Million population. To the best of our knowledge, this is the first report ever in this region where Xpert MTB/RIF method has been used for studying over all prevalence of MTB. This study is of great significance because it would provide baseline data in case if the scope is extended to other provinces in Pakistan.

Xpert MTB/RIF test is increasingly used in developing high burden countries to diagnose drug resistant MTB, while conventional test such as culture based DST is considered to be a “gold standard” for MDR-TB. In the present study, 389 (95.4 %) MTB isolates, found RIF resistant by Xpert MTB/RIF test, were confirmed to be resistant to RIF by DST. However the remaining 19 (4.6 %) isolates, initially diagnosed as resistant to RIF by Xpert MTB/RIF, were tested susceptible to RIF by DST. The previous reports indicates that lack of concordance in the results are not due to performance of molecular method [5, 15–18], but this discrepancy may be due to well-known fact that not every genotypic modification of *rpo*B gene affects phenotypic resistance to RIF correspondingly. It has been observed in the previous studies that the value of the RIF MIC strongly correlates with the mutation in *rpo*B RRDR. [19, 20] Feuerriegel et al. stated that in Sierra Leone, the isolates from re-treatment cases were re-tested by DNA sequencing, 5 out of 21 (24 %) cases were found RIF susceptible by DST. [21] In our view, the conventional test i.e. DST, fails to detect mutation in 19 (4.6 %) isolates. Present data indicates that these dissimilarities in mutation may be due to poor clinical scenario that is utmost common and undisputed, while few reports had previously suggested that such isolates have clinical relevance [22, 23].

The most common RRDR *rpo*B gene mutations within the 81 bp were detected in codons 531 (77 %), 513 (10.8 %), 526 (8.3 %), 511 (1.2 %), and 522 (1.5 %) sequences as selected by probes E, B, D, A, and C, respectively, using Xpert MTB/ RIF assay. A study by Yue et al. found these frequencies 531 (41 %), 526 (40 %), and 513 (4 %) in China [24]. Mboowa et al. also reported that 58 % mutations were in probe E in Kampala, Uganda [25]. Khan et al. also described mutations in codons 531 (52 %), 516 (15 %) and 526 (7.0 %) in Punjab, Pakistan [26]. These studies conclude that sequence 531 is the most prevalent codon related to RIF-resistance. According to the previous studies, the sensitivity and specificity of the MTB/RIF for detection of rifampicin resistance was 94.4-100 % and 98.3-100 % [5, 27].

In current study, we have reported 29 % MDR-TB in presumptive drug resistant TB patients in Khyber Pakhtoonkhwa, which is similar to Liu et al. finding as 30.4 % isolates were resistant to at least one first-line anti-TB drug [28]. Luiz et al. reported 44.3 % isolates resistant to at least one first-line anti-TB drug in Brazil which is more than our findings [29]. Ullah et al. [30] and Javaid et al. [11] reported 11.5 and 11.3 % resistance to at least one anti-TB drug in different parts of Punjab, Pakistan. In order to fight the risk MDR TB, a recent Drug Resistant Survey, Study was conducted in Pakistan showed that estimated percentage of MDR TB in new notified TB cases is 4.3 % and in Re-treatment cases is 19.4 % [1]. This difference may be due to geographical location, sample size, and methodology for selection of presumptive patients for MDR-TB.

WHO expanded its MDR-TB detection program in 2012 within the high TB burden countries and use of Xpert MTB/RIF Assay was approved for efficient detection of resistance against anti-TB drugs [1]. Thus the assay could be a useful tool in World’s fight against TB/MDR-TB especially in high TB burden countries such as Pakistan.

## Conclusions

In this report, the Xpert MTB/RIF assay detected the RIF-resistance associated mutations in RRDR 81 bp region. Hence, it is concluded from our study on a selected population, 29 % of patients had MDR-TB. Probe E related mutations (also known as codon 531and 533) were the most common *rpo*B genetic mutation [314 (77 %)], acknowledged by Xpert MTB/RIF assay. Least mutation was detected within the sequence 511 (1.2 %). Further studies ought to be done involving MDR-TB strains isolated from Pakistani population in order and data concerning these mutations would be helpful in development of novel therapies against TB disease.

## Abbreviation

CSF, cerebral spinal fluid; DST, drug sensitivity test; LRH, lady reading hospital; MDR-TB, multi drug resistant tuberculosis; PGMI, post graduate medical institute; PMDT, programmatic management of drug resistant tuberculosis; RIF; *rpo*B gene, RNA Polymerase B gene; RRDR, rifampicin resistant determinant region
